# Fluorescent probes for the visualization of membrane microdomain, deformation, and fusion

**DOI:** 10.1002/smo.20240059

**Published:** 2024-12-30

**Authors:** Pei‐Hong Tong, Tong‐Yuan Wu, Mingle Li, Hai‐Bin Wang, Feng Zheng, Lin Xu, Wei‐Tao Dou

**Affiliations:** ^1^ State Key Laboratory of Fine Chemicals College of Materials Science and Engineering Shenzhen University Shenzhen China; ^2^ Shanghai Key Laboratory of Green Chemistry and Chemical Processes Shanghai Frontiers Science Center of Molecule Intelligent Syntheses School of Chemistry and Molecular Engineering East China Normal University Shanghai China; ^3^ Department of Chemistry Korea University Seoul Korea; ^4^ College of Chemistry and Chemical Engineering Ningxia Normal University Guyuan China; ^5^ Wuhu Hospital East China Normal University (The Second People's Hospital, Wuhu) Wuhu China; ^6^ Chongqing Key Laboratory of Precision Optics Chongqing Institute of East China Normal University Chongqing China

**Keywords:** deformation, fluorescent probes, fusion, membrane microdomain

## Abstract

The cell membrane, a fluid interface composed of self‐assembled phospholipid molecules, is a vital component of biological systems that maintains cellular stability and prevents the invasion of foreign toxins. Due to its inherent fluidity, the cell membrane can undergo bending, shearing, and stretching, making membrane deformation crucial in processes like cell adhesion, migration, phagocytosis, and signal transduction. Within the plasma membrane are highly ordered dynamic structures formed by lipid molecules, known as “lipid rafts,” whose dynamic dissociation and reorganization are prerequisites for membrane deformation. Fluorescent probes have emerged as vital tools for studying these dynamic processes, offering a non‐destructive, in situ, and real‐time imaging method. By strategically designing these probes, researchers can image not only the microdomains of cell membranes but also explore more complex processes such as membrane fusion and fission. This review systematically summarizes the latest advancements in the application of fluorescent probes for cell membrane imaging. It also discusses the current challenges and provides insights into future research directions. We hope this review inspires further studies on the dynamic processes of complex cell membranes using fluorescent probes, ultimately advancing our understanding of the mechanisms underlying membrane dissociation, reorganization, fusion, and separation, and fostering research and therapeutic development for membrane‐associated diseases.

## Graphical abstract

Fluorescent probes have emerged as vital tools to image the microdomains of cell membranes and explore more complex processes such as membrane fusion and fission. This review systematically summarizes the latest advancements in the application of fluorescent probes for cell membrane imaging, aiming to our understanding of the mechanisms underlying membrane dissociation, reorganization, fusion, and separation, and fostering research and therapeutic development for membrane‐associated diseases.

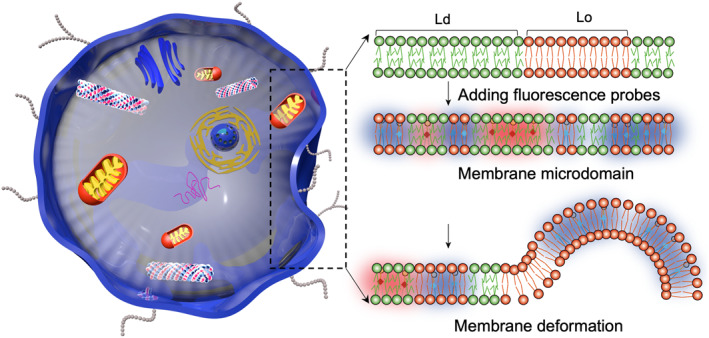

## 1 INTRODUCTION

**FIGURE 1:**
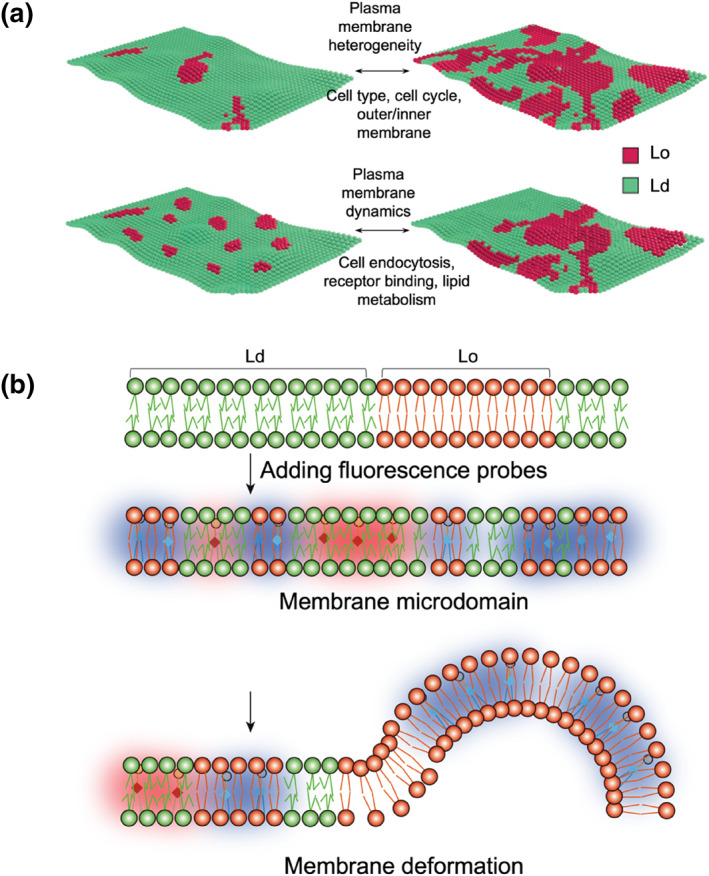
(a) Schematic diagram of cell membrane dynamics and heterogeneity arising from cellular physiological processes. Reproduced from Ref.^[2a]^ with permission. Copyright (2017), Springer Nature. (b) Basic schematic of fluorescent probes for imaging membrane microdomain and deformation.

The plasma membrane is a dynamic structure composed of a phospholipid bilayer and various embedded proteins.^[1]^ It not only serves as a fundamental cell barrier but also plays a critical role in cellular uptake, migration, proliferation, and signaling.^[2]^ Consequently, alterations in the structure, composition, and morphology of the plasma membrane reflect the physiological state of the cell, closely linked to major diseases such as cellular aging and cancer (Figure 1a).^[3]^ Therefore, developing imaging tools for the plasma membrane is of great practical value for understanding physiological processes associated with cell membranes.^[4]^

Among various analytical methods for studying plasma membranes,^[5]^ fluorescence analysis using probes has been widely used for real‐time in situ monitoring of cellular activities, metabolism, and intercellular communication on cell surfaces due to its high sensitivity, simplicity, and non‐invasiveness.^[6]^ Compared to fluorescent proteins,^[7]^ fluorescent dyes offer versatility, ease of synthesis, and excellent photostability, making them ideal for long‐term accurate imaging of plasma membranes.^[8]^ Following the initial use of fluorescent probes by Tasaki et al. (1968), in the study of membranes, the use of fluorescence to provide structural information at the microscopic level in biological membranes has become widespread.^[9]^ For instance, Klymchenko et al. worked on imaging cell membranes with proton and charge transfer dyes (3‐hydroxychromones, Nile Red, push‐pullfluorene, etc.), which changed their color and intensity in response to environment polarity.^[10]^ Goujon et al. had developed a series of mechanosensitive probes to visualize physical forces in cell membranes (fluorescent flippers).^[11]^ Next, Gupta et al. had developed a two‐component chemical tool called the HIDE probe, which enabled super‐resolution, long‐latency imaging of membrane dynamics.^[12]^ Other outstanding researchers have also made significant contributions to the development of the field of membrane probes.^[13]^

Current fluorescent dyes for cell membrane imaging are typically developed by binding fluorescent moieties to cytoplasmic membrane‐anchoring molecules, including alkyl chains, cholesterol, or cytoplasmic membrane protein tags, to provide these probes with better membrane anchoring properties and prolong their retention time at the plasma membrane. Meanwhile, the performance of fluorescent probes has an important impact on the results of cell membrane imaging. For example, the ideal fluorescent probe for super‐resolution imaging of the plasma membrane of living cells should have excellent photostability, high fluorescence quantum yield, good water solubility, and the ability to meet the specific requirements of the super‐resolution system (e.g., spontaneous scintillation or bursting under lossy light). In addition, near‐infrared (NIR) fluorescent probes^[14]^ are chosen to be able to penetrate deeper into the tissue layers. As such, fluorescent dyes can effectively track the structure and morphology of plasma membranes in real‐time during biological processes related to major diseases (Figure 1b).[^10^, ^15^]

This article reviews the progress of fluorescent probes in visualizing the plasma membrane. It summarizes the representative fluorescent probes reported in recent years and categorizes them based on microdomains, deformation, and fusion processes of the plasma membrane. In particular, it provides a detailed overview of the design principles, targeting mechanisms, and applications of fluorescent probes. Finally, it addresses the current challenges and potential future directions for using fluorescent probes in visualizing the plasma membrane.

## 2 FLUORESCENCE PROBES FOR IMAGING OF MEMBRANE MICRODOMAIN

**FIGURE 2:**
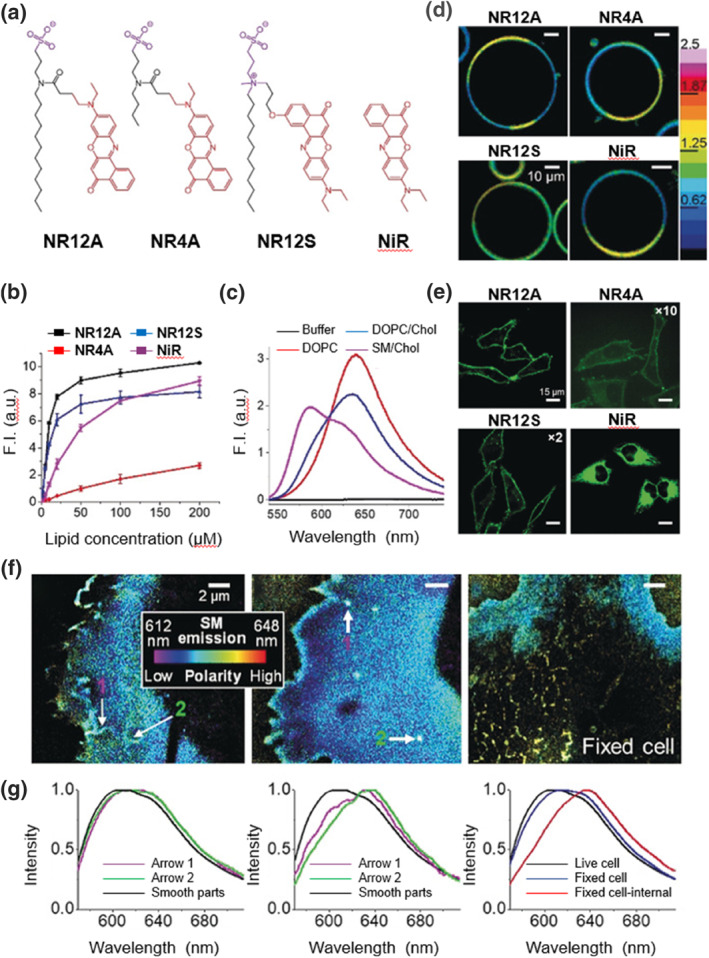
(a) Chemical structures of membrane probes **NR12A**, **NR4A**, **NR12S** and **NiR**. (b) Fluorescent intensity of probes **NR12A**, **NR4A**, **NR12S**, and **NiR** in the presence of DOPC LUVs. (c) Fluorescent intensity of probe **NR12A** in various solutions, including phosphate buffer solution (PBS), DOPC LUVs, DOPC/Chol LUVs, and SM/Chol LUVs. (d) Ratiometric CLSM images of DOPC/SM/Chol GUVs upon addition of probes **NR12A**, **NR4A**, **NR12S**, and **NiR**. (e) CLSM images of Hela cell lines incubated with probes **NR12A**, **NR4A**, **NR12S**, and **NiR**. (f) CLSM images of live and fixed COS‐7 cell lines labeled with **NR4A**. (g) Averaged single‐molecule spectra of tube‐like protrusions and smooth regions of the plasma membrane (left). Comparison of averaged single‐molecule spectra from tube‐like protrusions (left) and cluster structures (center) against smooth areas of the plasma membrane, along with spectra from live/fixed COS‐7 cell lines compared to internal membranes of fixed cells (right). Reproduced from Ref.^[^ ^17^ ^]^ with permission. Copyright (2019), John Wiley and Sons. CLSM, confocal laser scanning microscopy; GUVs, giant unilamellar vesicles; LUVs, large unilamellar vesicles.

Membrane deformation and fusion are closely related to the dynamic changes between ordered and disordered phases of unsaturated phospholipid molecules.^[16]^ Although many membrane probes have been developed, very few can effectively study the dynamic processes occurring within the microdomains of cell membranes. With the introduction of sulfonate anions and modification of alkyl chains of varying lengths, Danylchuk et al. reported a series of exceptional membrane probes,^[17]^ **NR4A**, **NR12A** and **NR12S** (Figure 2a). As shown in Figure 2b, these probes exhibited significant fluorescence enhancement when incorporated into large unilamellar vesicles (LUVs) composed of dioleoylphosphatidylcholine (DOPC). As a result of its shorter alkyl chain, **NR4A** showed the weakest fluorescence enhancement. In contrast, the tightly packed ordered phase provided strong hydrophobic interactions, resulting in marked fluorescence enhancement for **NR12A** in liquid‐ordered (Lo) LUVs, such as those made from DOPC or DOPC/cholesterol mixtures. Notably, **NR12A** exhibited a blue shift of approximately 50 nm in liquid‐disordered (Ld) LUVs formed from egg sphingomyelin (SM) and cholesterol mixtures, reflecting the lower local polarity and hydration of the Lo phase (Figure 2c). A two‐color confocal laser scanning microscopy (CLSM) study revealed that **NR12A** and **NR4A** could differentially image membrane microdomains in LUVs composed of DOPC/SM/cholesterol (1:1:0.7), which contained both Lo and Ld phases (Figure 2d). Probes with long alkyl chains were able to insert into the cell membrane without apparent internalization (Figure 2e), unlike those with shorter or no alkyl chains. **NR4A**'s shorter alkyl chain allows for reversible membrane binding, making it particularly suitable for high‐resolution stimulated emission depletion (STED) microscopy. By combining the fluorescence spectra and positions of individual **NR4A** molecules, nanoscale spatial and minute‐level temporal resolution imaging of local lipid order can be achieved, with intensity‐weighted average emission wavelength presented as pseudocolor. In CLSM images of live COS‐7 cells labeled with **NR4A**, color changes from blue to cyan were observed, along with distinct membrane protrusions and invaginations, potentially associated with lipid rafts (Figure 2f). Additionally, **NR4A** penetrated the membranes of organelles, resulting in a red shift in fluorescence that indicates reduced lipid order in these organelles (Figure 2g). Through the rational design of fluorescent probes to modulate their interactions with phospholipid molecules, this study successfully developed probes for super‐resolution microscopy of membrane microdomains. This method provides an opportunity to study lipid order heterogeneity concerning membrane topology at the nanoscale.

**FIGURE 3:**
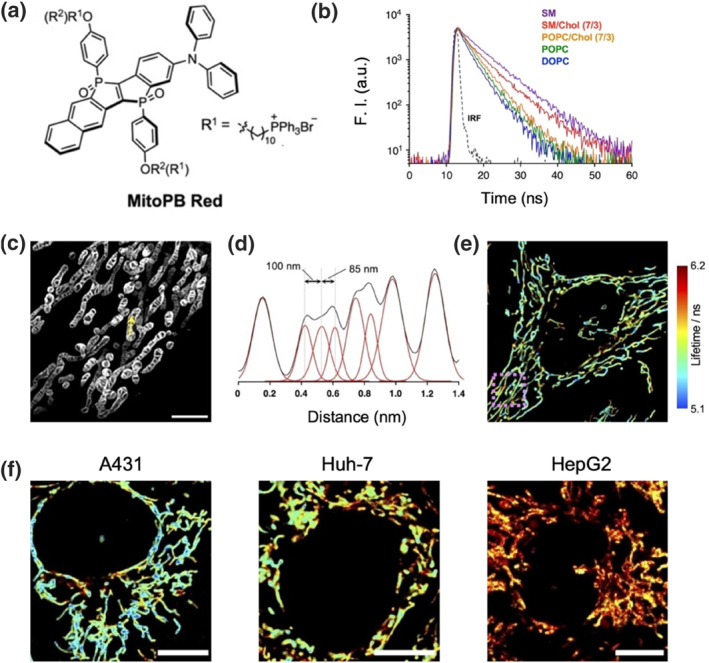
(a) Chemical structure of the membrane probe **MitoPB Red**. (b) Fluorescence decay of **MitoPB Red** upon the addition of various LUVs containing different phospholipid molecules, including SM, SM/Chol, POPC/Chol, POPC, and DOPC. (c) STED images of mitochondria in Hela cell lines stained with **MitoPB Red**. (d) Fluorescent profile (blank) and curve‐fitting (red) from the arrow of (c). FLIM images of mitochondria labeled with **MitoPB Red** in (e) Hela and (f) other cell lines (A431, Huh‐7, and HepG2). Reproduced from Ref.^[^ ^13b^ ^]^ with permission. Copyright (2019). National Academies Press. FLIM, fluorescence lifetime iamging microscopy; LUVs, large unilamellar vesicles; STED, stimulated emission depletion.

Cell membranes also act as a barrier against external environmental disturbances that may interfere with various intracellular organelles. Similarly, these sub‐organelles are enclosed by plasma membranes composed of phospholipids. A particular feature of mitochondria is their structure, which includes a relatively flat outer mitochondrial membrane and a highly convoluted inner mitochondrial membrane.^[18]^ The dynamic characteristics of the mitochondrial membrane are essential for understanding the unique functions associated with this organelle.^[19]^ Recently, Wang et al. reported a mitochondrial membrane fluorescent probe, **MitoPB Red**, which consists of phospholo[3,2‐*b*]phosphole‐*P*, *P*′‐dioxide scaffolds and incorporates triphenylphosphonium (TPP) to target mitochondrial membranes (Figure 3a).^[13b]^ **MitoPB Red** emits red fluorescence and exhibits distinct variations in fluorescence lifetime between Lo and Ld liposomes. As shown in Figure 3b, 1,2‐dioleoyl‐sn‐glycero‐3‐phosphocholine (DOPC), and 1‐ palmitoyl‐2‐oleoyl‐phosphatidylcholine (POPC), which contain more unsaturated double bonds than SM, tend to form loosely packed Ld phases that increase water permeability and accelerate non‐radiative decay in the excited state (ES), thereby reducing fluorescence lifetime. Consequently, the probe is capable of detecting lipid order through fluorescence lifetime imaging. In STED imaging**, MitoPB Red** provided high‐resolution images of mitochondrial cristae, with significantly higher fluorescence intensities observed in the inner membrane compared to the outer membrane (Figure 3c). This suggests a degree of inner membrane selectivity, possibly due to the presence of unsaturated cardiolipin (CL), which contributes to lower order and increased fluidity. Moreover, spectral deconvolution processing allowed clear identification of individual cristae distribution within the inner membrane (Figure 3d). Fluorescence lifetime imaging revealed significant heterogeneity in mitochondrial imaging within HeLa cells, which correlates with the highly dynamic nature of mitochondria (Figure 3e). Additionally, different cell types exhibited distinct characteristics of the inner membrane. In A‐431 (human skin cancer), HepG2 (human liver cancer), and Huh‐7 (hepatocellular carcinoma) cells, fluorescence lifetimes significantly increased, possibly due to elevated cholesterol levels in mitochondria resulting from lipid metabolism processes in liver cells (Figure 3f). This study developed an effective tool for investigating membrane dynamics at the sub‐organelle level, offering new insights into the processes of organelle fusion and fission driven by lipid distribution changes during physiological or pathological events.

**FIGURE 4:**
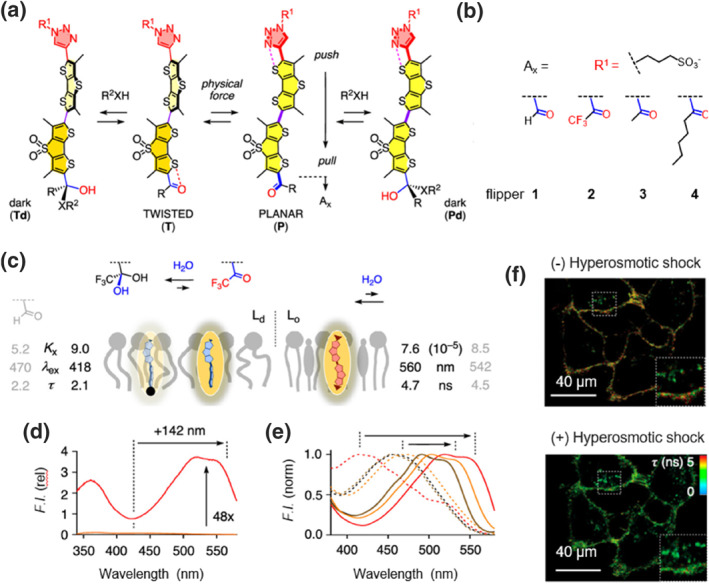
(a) Proposed mechanism of flipper probes for detecting membrane dynamics in response to different stimuli. (b) Chemical structures of the substituent groups in flipper **1**–**4**. (c) Schematic representation of the detection mechanism for Lo or Ld LUVs using flipper 2 (blank) in comparison to flipper 1 (gray). (d) Excitation spectra of flipper **2** in DOPC and SM/CL LUVs. (e) Normalized excitation spectra of flipper **1**–**4** (orange for **1**, red for **2**, brown for **3**, and black for **4**) in DOPC (solid) and SM/CL (dashed) LUVs. (f) FLIM images of Hela cells treated with hyperosmotic shock, labeled with flipper **2**. Reproduced from Ref.^[^ ^21^ ^]^ with permission. Copyright (2020), American Chemical Society. FLIM, fluorescence lifetime iamging microscopy; LUVs, large unilamellar vesicles.

Although utilizing cis–trans isomerization driven by intramolecular rotation has enabled super‐resolution imaging of cell membranes, a single switching process is insufficient to capture the complex deformation dynamics arising from the heterogeneity of membranes. Therefore, developing super‐resolution imaging techniques^[20]^ regulated by multiple factors is essential. Based on their previously reported Flipper probes, Garcia‐Calvo et al. combined mechanosensitive chalcogen‐bonded cascade switching with dynamic covalent carbonyl chemistry to create super‐resolution fluorescent probes (**Flipper 1**–**4**) responsive to both mechanical force and polarity.^[21]^ Notably, the probe undergoes ultrafast chalcogen‐bonded cascade switching in the ES, facilitating the transition from a twisted to a planar configuration, which results in reduced fluorescence. A reversible conversion between hemiacetal and hemithioacetal structures is triggered due to the dynamics of lipids within the membrane, enabling synergistic super‐resolution imaging (Figures 4a and 4b). In LUVs with different lipid compositions, **Flipper 2** exhibited significantly stronger fluorescence in Lo compared to Ld phase, attributed to increased conjugation as the compound transitioned from a twisted to planar form (Figure 4c,d). Specifically, **Flipper 2** displayed more pronounced redshifts and fluorescence intensity increases than **Flipper 2**, **3**, and **4**, which contain a trifluoromethyl ketone group that is more susceptible to nucleophilic addition, thereby disrupting the push‐pull system and facilitating planarization (Figure 4e). The fluorescence lifetime of the probe significantly decreased with increased osmotic pressure in HeLa cells, allowing the imaging of membrane tension in living cells (Figure 4f). This study provides a method for constructing membrane probes that integrate mechanical‐sensitive cascade switching with dynamic covalent carbonyl chemistry, thereby aiding in the investigation of dynamic processes in cell membranes under multiple synergistic stimuli.

**FIGURE 5:**
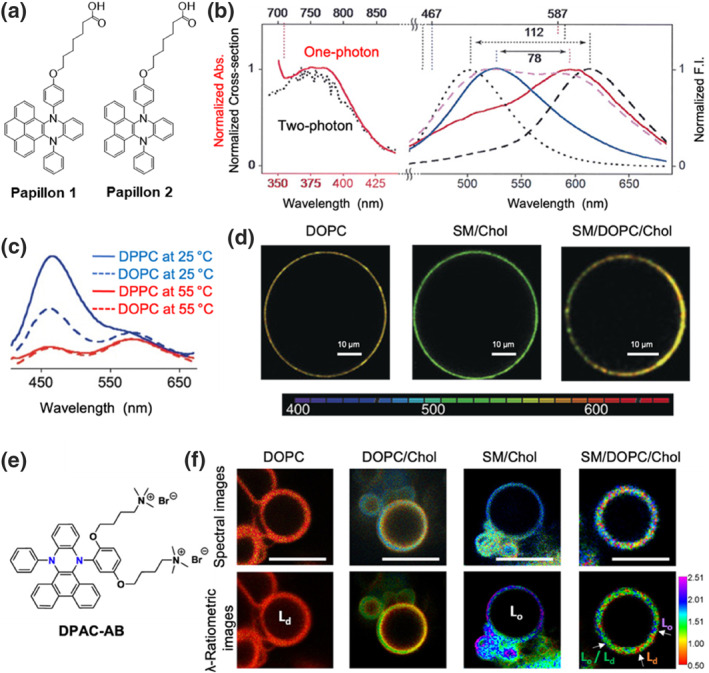
(a) Chemical structures of **Papillon 1** and **2**. (b) Normalized (left) one‐photon, two‐photon absorption, and (right) fluorescence intensity of **Papillon 2** in different solvents, including H_2_O (black, dotted), EtOAc (black, dashed), DPPC LUVs at 25°C (blue, solid), DOPC LUVs at 25°C (magenta, solid) and DPPC LUVs at 25°C (red, solid). (c) Not‐normalized emission of **Papillon 1** in different LUVs at 25 and 55°C. (d) TPEFM images of GUVs composed of DOPC, SM/Chol, or SM/DOPC/CL labeled with **Papillon 2**. (e) Chemical structure of **DPAC‐AB**. (f) *λ*‐Ratiometric fluorescent microscopy images of GUVs composed of DOPC, SM/Chol, or SM/Chol, or SM/DOPC/CL labeled with **DPAC**‐**AB**. Reproduced from Ref.^[^ ^24^ ^]^ with permission. Copyright (2018), John Wiley and Sons. Reproduced from Ref.^[^ ^25^ ^]^ with permission. Copyright (2020), American Chemical Society. DOPC, dioleoylphosphatidylcholine; GUVs, giant unilamellar vesicles; LUVs, large unilamellar vesicles; SM, sphingomyelin; TPEFM, two‐photon excitation fluorescence microscopy.

Molecular rotors and mechanosensitive flipper probes are traditionally employed for membrane detection by restricting the free rotation of the probe through interactions with local phospholipid molecules.[^6^, ^22^] Recently, *N*,*N*′‐diaryl‐dihydrodibenzo[*a*,*c*]phenazines (DPACs) have emerged as a unique class of fluorescence probes with single‐excitation and two‐emission properties. In the ES, these probes undergo a rare transition from a bent to a planar configuration, elongating the *π*‐delocalization over the benzo[*a*,*c*]phenazines core and resulting in dramatic Stokes shift emissions, termed vibration‐induced emission (VIE).^[23]^ When environmental factors hinder the planarization process of VIE‐based fluorophores, the red emission decreases while the blue emission increases, allowing for the detection of changes in viscosity, polarity, and temperature. Based on this principle, Humeniuk et al. designed and synthesized the VIE‐based ratiometric membrane probes (**Papillon 1** and **2**), which incorporate hydrophilic carboxyl groups and hydrophobic VIE units (Figure 5a).^[24]^ Compared to classical DPAC motifs (**Papillon 1**), **Papillon 2**, which contains a pyrene moiety, exhibits a significant red shift in both absorption and emission spectra (Figure 5b) attributed to the increased conjugation of the excited‐state molecule. In giant unilamellar vesicles (GUVs) composed of lipids with varying degrees of saturation, the **Papillon** probes displayed distinct lipid order selectivity. A typical blue emission is observed in solid‐ordered (So) dipalmitoyl phosphatidylcholine (DPPC) GUVs, where Papillon's free molecular conformation is constrained. In disordered lipids, however, relaxed conformational constraints allowed the excited‐state molecules to planarize more readily, resulting in the characteristic red emission (Figure 5c), consistent with two‐photon excitation fluorescence microscopy imaging (Figure 5d). The undesirable hydrophobicity of the Papillon limited its application in biological membranes. Zhang et al. addressed this issue by incorporating quaternary ammonium salts into the structure, creating the membrane probe **DPAC‐AC** with distinct hydrophobic and hydrophilic features (Figure 5e).^[25]^ This modification has enabled its application in the dynamic analysis of plasma membrane viscosity, providing new tools for the dynamic visualization of cell membranes (Figure 5f). Due to its unique ratiometric fluorescence properties, this probe holds promise for investigating more complex membrane deformation processes such as cell division and fusion.

**FIGURE 6:**
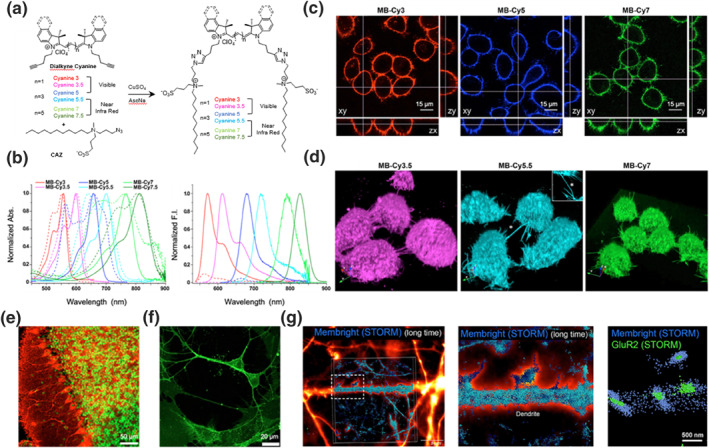
(a) Chemical structures and synthetic routes of membrane probes **MB‐Cy3**, **MB**‐**Cy3**.**5**, **MB‐Cy5**, **MB‐Cy5.5**, **MB‐Cy7** and **MB‐Cy7.5**. (b) Normalized absorption (left) and emission (right) spectra of the probes with (solid lines) and without (dashed lines) the addition of DOPC LUVs. (c) CLSM images of KB cell lines after incubation of membrane probes **MB‐Cy3**, **MB**‐**Cy5**, and **MB‐Cy7**. (d) Reconstructed 3D CLSM images of KB cell lines after incubation with membrane probes **MB‐Cy3.5**, **MB‐Cy5.5**, and **MB‐Cy7**. (e) CLSM images of brain slices stained with **MB‐Cy3.5**. (f) CLSM images of neuronal cells incubated with **MB‐Cy5**. (g) 3D STORM images of hippocampal neurons labeled with **MB‐Cy3.5**. Reproduced from Ref.^[^ ^27^ ^]^ with permission. Copyright (2019), Elsevier. CLSM, confocal laser scanning microscopy; LUVs, large unilamellar vesicles; STORM, stochastic optical reconstruction microscopy.

The majority of common cell membrane probes emit fluorescence in the visible light range, resulting in significant background interference and a limited depth of penetration.[^8^, ^26^] Collot et al. addressed this issue by developing NIR cell membrane probes, replacing the indole moiety with a benzo[e]indole group, extending the polymethine chain length, and red‐shifting the emission wavelength (Figure 6a).^[27]^ Typical cyanine absorption and fluorescence spectra were observed in LUVs composed of DOPC. The **MemBright** (**MB**)**‐Cy3**, **MB**‐**Cy3**.**5**, and **MB‐Cy5** probes emit in the visible range, while the **MB‐Cy5.5**, **MB**‐**Cy7**, and **MB‐Cy7.5** emit in the NIR‐I region (Figure 6b). KB human cells, known for their regular shape, were selected for imaging studies. Vertical projections of the CLSM images demonstrated that these probes successfully inserted the cell membrane without causing significant internalization (Figure 6c). Furthermore, 3D CLSM reconstructions revealed tunneling nanotubes that may facilitate the exchange of information and materials between cells, thus enabling the visualization of intercellular communications (Figure 6d). While longer methylene chains improve tissue penetration but also compromise probe stability, leading to photobleaching, which hinders prolonged high‐resolution imaging. Notably, the probe **MB‐Cy3.5** was capable of labeling neurons within mouse brain slices and isolated neurons with high selectivity, allowing for the visualization of distinct neuronal regions in the brain (Figure 6e). Its excellent neuronal imaging capabilities also enable its use in long‐term stochastic optical reconstruction microscopy and imaging of dendritic processes, clearly revealing axonal wrapping around dendrites (Figure 6f,g), providing a reliable imaging tool for studying critical signaling pathways in the brain. Nevertheless, the inherent instability of the cyanine groups can lead to degradation or photobleaching of the probe. If the stability issues can be addressed, these biomembrane probes could become powerful tools for biomembrane imaging in the field of cell biology.

**FIGURE 7:**
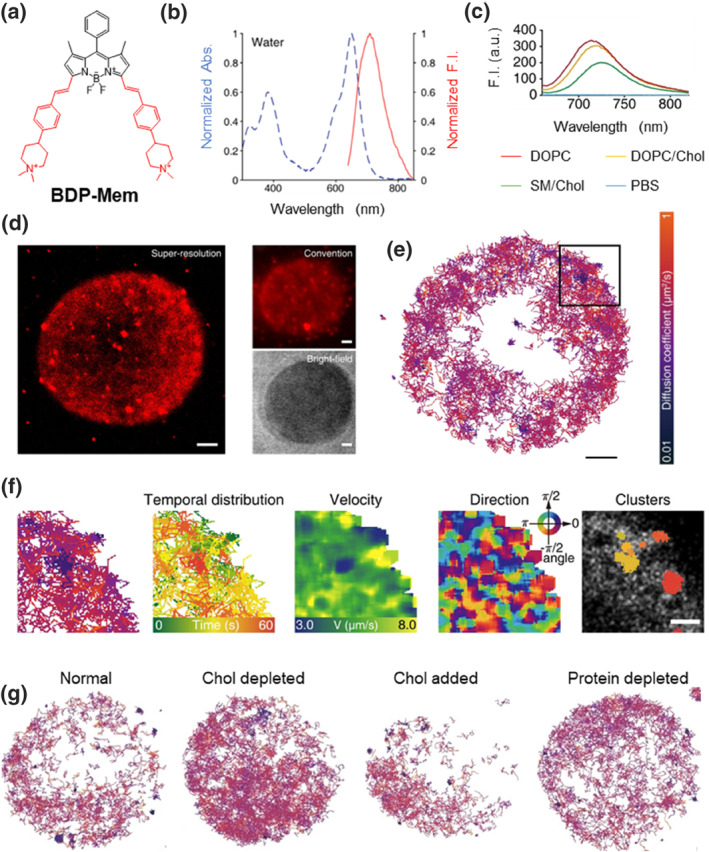
(a) Chemical structure of the membrane probe **BDP‐Mem**. (b) Normalized UV‐Vis‐NIR absorption and fluorescence emission spectra of **BDP‐Mem** in H_2_O. (c) Fluorescence intensity of **BDP‐Mem** in the presence of PBS or LUVs composed of different phospholipids (DOPC, DOPC/Chol, and SM/Chol). (d) Super‐resolution and convention imaging of a red blood cell labeled with **BDP‐Mem**. (e) Overlay of single‐molecule tracking trajectories of **BDP‐Mem** on the membrane of a red blood cell. (f) Enlarged view of the boxed region in panel (e), depicting tracking trajectories, temporal distribution, velocity, direction, and clusters. (g) Overlay of single‐molecule tracking trajectories of **BDP‐Mem** on the membrane of a red blood cell under different treatments: normal, Chol depleted, Chol added and protein depleted. Reproduced from Ref.^[^ ^15f^ ^]^ with permission. Copyright (2022), John Wiley and Sons. DOPC, dioleoylphosphatidylcholine; LUVs, large unilamellar vesicles; PBS, phosphate buffer solution; SM, sphingomyelin.

Various erythrocyte membrane disorders are believed to be closely linked to dysfunctions in the erythrocyte membrane.^[28]^ Understanding the ultrastructure and lipid rearrangement dynamics of the erythrocyte membrane may help uncover the underlying causes of these dysfunctions. Ye et al. reported a BODIPY‐based membrane probe (**BDP‐Mem**) featuring a cis‐trans isomerized *p*‐piperazinyl styryl unit that causes fluorescence blinking for high‐resolution fluorescence imaging (Figure 7a).^[15f]^ With a maximum absorption peak at 660 nm and a NIR fluorescence peak at 700 nm, **BDP‐Mem** enhances deep‐tissue fluorescence imaging due to its moderate cell penetration (Figure 7b). In LUVs composed of phospholipids with varying saturation, **BDP‐Mem** preferentially binds to disordered LUVs, resulting in a blue shift in fluorescence. This shift occurs because the hydrophobic environment of disordered lipids suppresses the non‐radiative relaxation of the probe (Figure 7c). The probe's cis–trans isomerization allows for super‐resolution fluorescence imaging of erythrocytes, revealing significant differences compared to traditional imaging methods (Figure 7d). Long‐term imaging based on single‐molecule tracking trajectories of **BDP‐Mem** on the membrane of a red blood cell demonstrated spatiotemporal heterogeneity on the membrane surface (Figure 7e). Additionally, a detailed analysis of the local time trajectory, spatial trajectory, and vector velocity of the probe confirmed that membrane microdomains are highly heterogeneous (Figure 7f). The content of cholesterol and membrane proteins in the cell membrane influences its fluidity. Single‐molecule trajectory analysis showed that decreasing cholesterol concentration increased membrane fluidity, whereas variations in membrane protein concentration did not significantly impact membrane heterogeneity (Figure 7g). It is likely that trypsin affects proteins on the outer surface of the cell membrane without influencing those embedded within it. This study further highlights that the dynamic rearrangement of lipid molecules is crucial for membrane proteins to perform their biological functions. It provides a valuable tool for systematically evaluating dynamic changes in erythrocyte membrane structure, offering insights into the mechanisms of erythrocyte membrane disorders and potentially aiding in the development of therapeutic interventions.

## 3 DEFORMATION OF THE CYTOPLASMIC MEMBRANES

Cytoplasmic membrane deformation can result from changes in the intra‐ and extracellular environment, cell movement, intracellular protein transport, and secretion. These deformations can take various forms, including expansion, contraction, bending, and rupture.[^16^, ^29^] Such changes enable cells to perform essential physiological functions like material exchange, intracellular transport, and morphological adaptations. Therefore, observing cytoplasmic membrane deformation is crucial for understanding cell biological processes, and disease mechanisms, and developing new therapeutic approaches.

**FIGURE 8:**
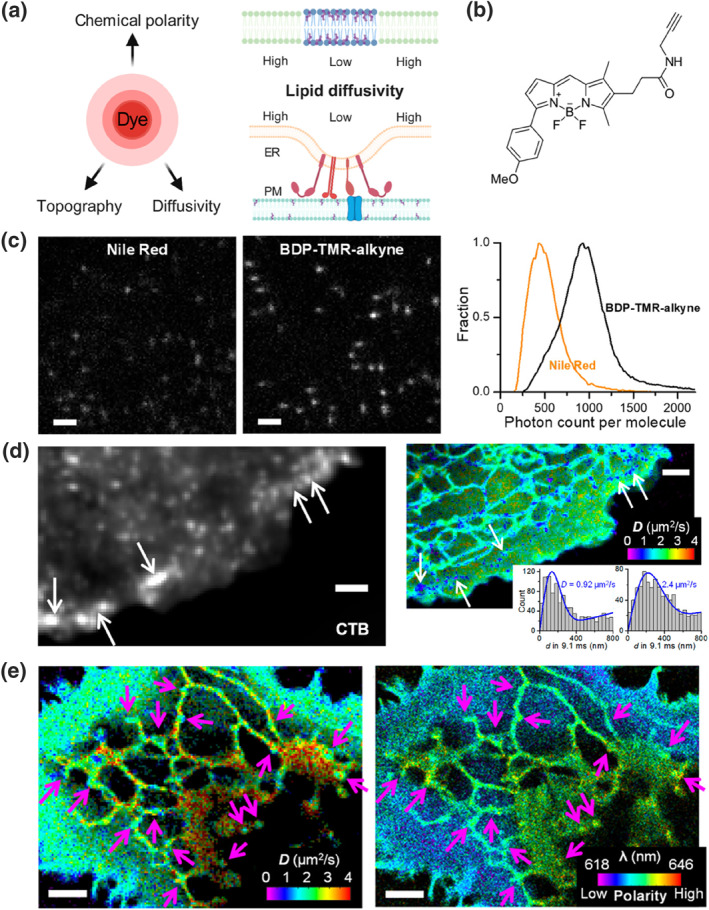
(a) Nanoscale diffusion heterogeneity in cell membranes investigated using multidimensional single‐molecule and super‐resolution microscopy. (b) Chemical structure of **BDP‐TMR‐alkyne**. (c) Comparison of single‐molecule membrane probes and their single photon count distributions. (d) Stain‐labeled cholera toxin B superimposed on the fluorescence image of a COS‐7 cell, along with the **BDP‐TMR‐alkyne** SMdM of the same cell (inset: Typical single‐molecule displacement distributions for low diffusivity nanodomains [left] versus the rest of the plasma membrane [right] and its maximum likelihood estimation fit [blue curve]). (e) Simultaneous images of the principal‐direction SMdM and the single‐molecule localization microscopy taken in living COS‐7 cells. Reproduced from Ref.^[^ ^30^ ^]^ with permission. Copyright (2020), American Chemical Society. SMdM, single‐molecule displacement/diffusivity mapping.

Fluorescent probes are valuable tools for visualizing cytoplasmic membrane deformation, offering several advantages: (1) They provide excellent sensitivity and resolution, capable of detecting subtle membrane deformations. (2) They allow for real‐time imaging of membrane deformation processes, which is vital for studying dynamic changes and the molecular mechanisms underlying these changes. (3) They typically do not damage the cell membrane during detection, enabling studies under near‐physiological conditions. Continuous fine mapping of local diffusivity in cell membranes could enhance our understanding of inherent regularities, yet challenges persist. To address this, Yan et al. exploited the transient intramembrane diffusion of single probe molecules for imaging nanoscale topography, achieving single‐molecule displacement/diffusivity mapping for cellular membranes (Figure 8a).^[30]^ Firstly, **BDP‐TMR‐alkyne** (Figure 8b), a water‐soluble dye with a high affinity for lipid bilayers, was selected as the plasma membrane probe. Upon excitation at 560 nm, **BDP‐TMR‐alkyne** exhibited a fluorescence turn‐on rate approximately 10 times higher in the organic phase than in aqueous media. Compared to Nile Red, **BDP‐TMR‐alkyne** demonstrated a higher fluorescence quantum yield (95%) and extinction coefficient, resulting in greater brightness and superior single‐molecule imaging (Figure 8c). By accumulating the positions and transient displacements of >106 probe molecules, **BDP‐TMR‐Alkyne** provided continuous single‐molecule imaging of cell membranes under stroboscopic excitation, facilitating super‐resolution topography and diffusion mapping (Figure 8d,e). The study revealed that the degree of protein crowding and lipid accumulation primarily determines cytoplasmic membrane diffusivity. This research marks the first investigation of molecular diffusion in cellular membrane systems at the nanoscale using **BDP‐TMR‐alkyne**, highlighting the multiple factors involved in regulating membrane diffusion and deformation.

**FIGURE 9:**
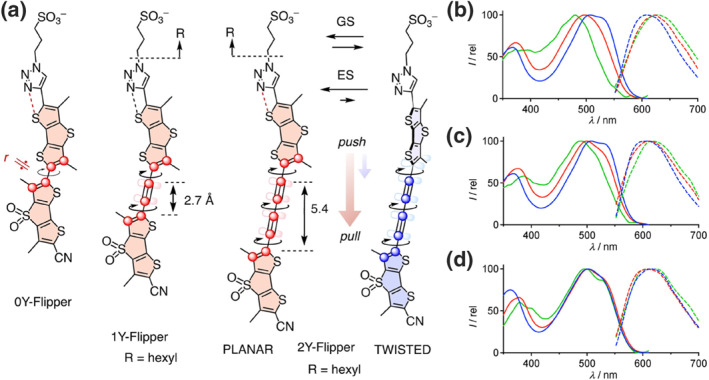
(a) Comparison of **1Y‐Flipper** and **2Y‐Flipper** with the **0Y‐Flipper**, highlighting the associated p‐orbitals (lobes, circles), rotatable bonds (curved arrows), distances in Å (double‐headed arrows), repulsion (*r*), conformational equilibria of the ground (GS) and excited state (ES), along with push‐pull dipoles (downward arrows). Normalized smoothed excitation (solid line) and emission (dashed line) spectra of the **2Y‐Flipper** (green), **1Y‐Flipper** (red) and **0Y‐Flipper** (blue) in (b) DPPC, (c) 1,2‐distearoyl‐sn‐glycero‐3‐phospho choline (DSPC), and (d) 1,2‐dibehenoyl‐sn‐glycero‐3‐phospho‐choline (DBPC) LUVs. Reproduced from Ref.^[^ ^32^ ^]^ with permission. Copyright (2024), John Wiley and Sons. LUVs, large unilamellar vesicles.

In addition to cytoplasmic membrane diffusion, external pressure or stretch can also lead to deformation of the cell membrane, resulting in changes in its thickness.^[31]^ These thickness variations affect the membrane's permeability and stability, which in turn influence the exchange of substances between the cell and its environment. To study and track the dynamic behavior of plasma membranes, particularly thicker membranes, Pamungkas et al. reported a series of novel fluorescent probes based on core‐alkynylated fluorescent flippers, including **0Y‐Flipper**, **1Y‐Flipper**, and **2Y‐Flipper** (Figure 9a).^[32]^ Compared to **0Y‐Flipper**, the **1Y‐Flipper** and **2Y‐Flipper** feature enhanced photophysical properties due to the significant extension of the rigid scaffold, increased conjugation, reduced core repulsion, and improved core rotation. These modifications facilitate selective imaging in thick hydrophobic membranes. In membranes of varying thickness (DPPC < DSPC < DBPC), the binuclear alkynylated **2Y‐Flipper** selectively imaged unusually thick higher‐order membranes, demonstrating higher intensity excitation maximum redshifts and sharper vibrational structures compared to **0Y‐Flipper** and **1Y‐Flipper** (Figure 9b–d). Depending on the environment, **2Y‐Flipper** can function as a flipper limb, a rotor, or a combination of both. The ability of **2Y‐Flipper** to embed and stabilize within thick membranes while maintaining the sensitivity of its fluorescent properties enables real‐time monitoring and tracking of dynamic changes in these thick membranes. By providing responsive trackers for membranes of different thicknesses, this research addresses a longstanding challenge in chemical biology, opening up promising avenues for bioimaging.

**FIGURE 10:**
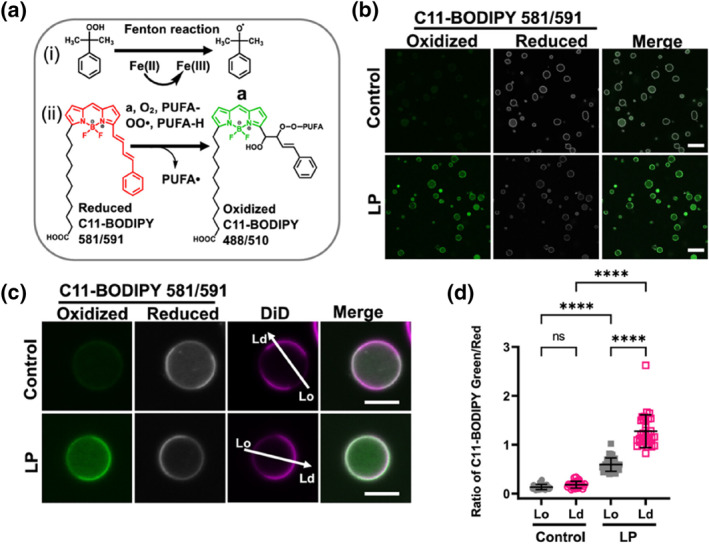
(a) Proposed mechanism by which PUFA peroxyl radicals react with **C11‐BODIPY** 581/591, leading to deconjugation and a blue shift in emission wavelength to 510 nm. (b) GPMVs were either untreated or incubated with Fe(II) and H_2_O_2_ to induce lipid peroxidation, followed by labeling with **C11‐BODIPY**. (c) Representative images of individual GPMVs labeled with **C11‐BODIPY** under control conditions and post‐lipid peroxidation. (d) The ratio of green (oxidized) to red (reduced) BODIPY 581/591 fluorescence in ordered versus disordered structural domains under control and peroxidation conditions. Reproduced from Ref.^[^ ^34^ ^]^ with permission. Copyright (2024), American Chemical Society. GPMVs, giant plasma membrane vesicles.

Moreover, free radical oxidation of cytoplasmic membranes leads to thinning of the phospholipid bilayer, altered fluidity, and increased membrane permeability in model systems.^[33]^ Balakrishnan et al. utilized cell‐derived giant plasma membrane vesicles (GPMVs) as a model to study the effects of lipid peroxidation on ordered membrane domains, often referred to as membrane rafts.^[34]^ To detect lipid peroxidation, C11‐BODIPY 581/591 (**C11‐BODIPY**) was used as a fluorescent ratio probe. Under conditions of free radical‐induced oxidation, its fluorescence properties change from red (590 nm) to green (510 nm) enabling ratiometric imaging of plasma membrane peroxidation at the cellular level (Figure 10a,b). When GPMVs were treated with free radical reagents, the fluorescence signal of reduced **C11‐BODIPY** (red) decreased, while the fluorescence signal of oxidized **C11‐BODIPY** (green) increased. This indicated that plasma membrane peroxidation significantly enhanced the phase separation tendency of GPMVs, resulting in the coexistence of liquid‐ordered and liquid‐disordered membrane domains, as well as an increased relative abundance of the disordered phase (Figure 10c). Notably, the green/red fluorescence ratio in the disordered structural domains was significantly higher in the treated GPMVs (Figure 10d). This suggests that the level of lipid peroxidation in the disordered domains is greater than that in the ordered domains. However, the extent to which plasma membrane peroxidation affects cell structure and morphology remains to be determined.

**FIGURE 11:**
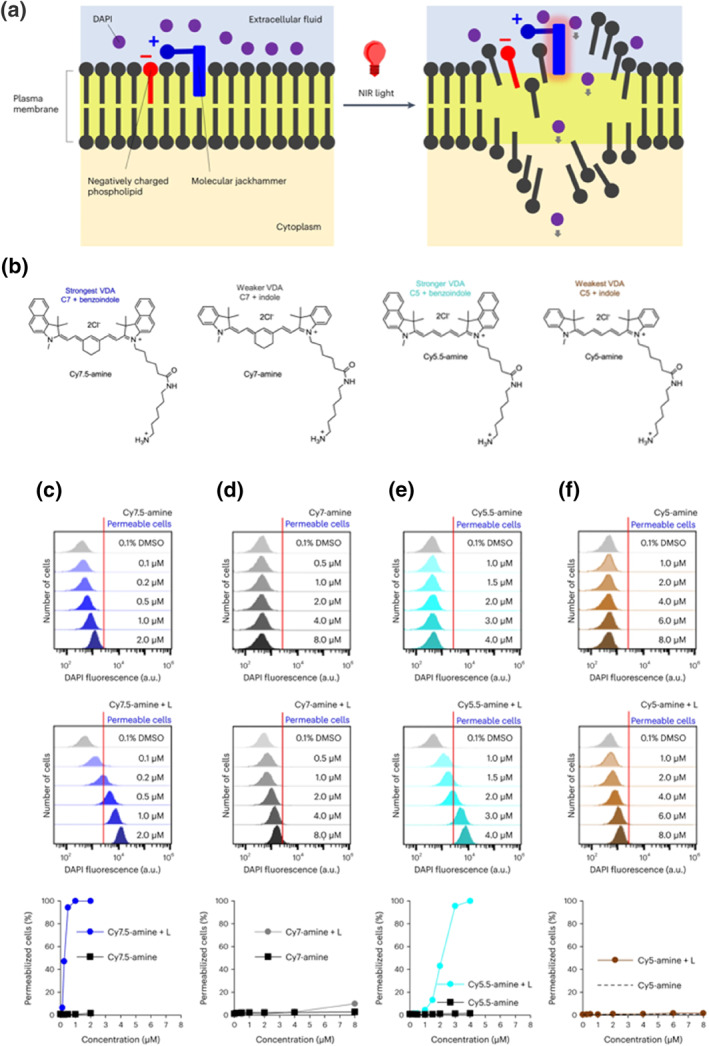
(a) Proposed mechanism for the opening of cell membranes by cell‐associated molecular jackhammers (MJH). (b) Chemical structures of **Cy7.5‐amine**, **Cy7‐amine**, **Cy5.5‐amine**, and **Cy5‐amine**. Effects of different molecular structures on molecular‐mechanical interactions in cell membrane deformation: (c) **Cy7.5‐amine**, (d) **Cy7‐amine**, (e) **Cy5**.**5**‐**amine**, and (f) **Cy5‐amine**. Reproduced from Ref.^[^ ^35^ ^]^ with permission. Copyright (2024), Springer Nature.

In the previous examples, probes were used to observe the dynamic deformation of the cell membrane. However, fluorescent probes can also vibrate at specific frequencies under light conditions, leading to a deformation‐to‐rupture process in the cytoplasmic membrane. By employing NIR light to drive the vibration of cell membrane‐associated aminocyanines, Ayala‐Orozco et al. induced rapid cytoplasmic membrane deformation and rupture within a very short time frame (Figure 11a).^[35]^ Specific structural features of the aminocyanines, such as the extension of the conjugated structure or modifications to the aromatic ring, directly influence the molecular mechanistic motion of these probes (**Cy7.5‐amine**, **Cy7‐amine**, **Cy5.5‐amine**, **Cy5‐amine**) (Figure 11b). As illustrated in Figure 11c–f, longer conjugation lengths of paraformaldehyde bridges and extensions of aromatic rings improve the efficiency of molecular mechanistic interaction, and **Cy7.5‐amine** demonstrated increased cellular penetration when NIR light induces Vibronic‐driven action (VDA). This indicates that excitation of the vibronic shoulder of **Cy7.5‐amine** at 730 nm, along with extensive conjugation of the aryl ring in the benzoindoles, is essential for maximizing VDA. Additionally, the strength of the VDA molecular mechanical interactions is closely associated with the molecular structure. These findings reveal a novel mechanical interaction at the molecular level that can lead to cytoplasmic membrane rupture and deformation, potentially facilitating the elimination of cells or enhancing the efficacy of adjuvant therapeutic cells.

## 4 FUSION OF CYTOPLASMIC MEMBRANES

**FIGURE 12:**
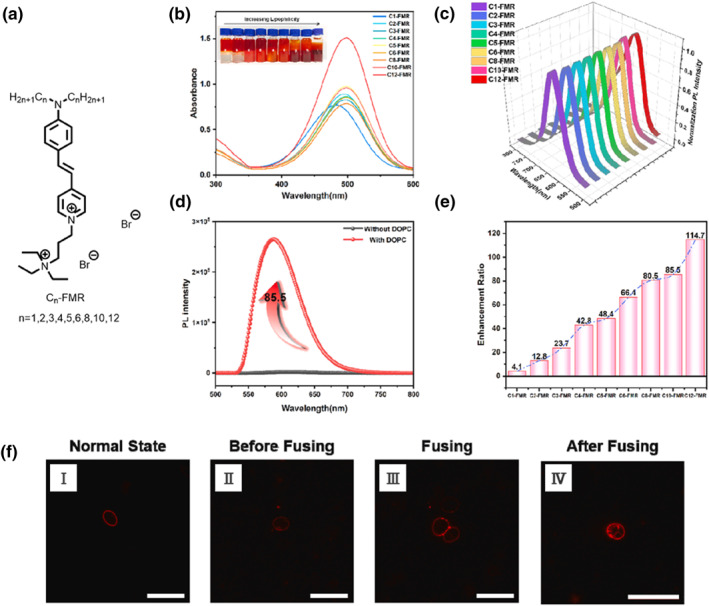
(a) Chemical structures of **C**_**n**_ **‐FMR**. (b) UV‐visible absorption spectra of FMR molecules in dimethyl sulfoxide (DMSO). Inset: Qualitative illustration of the partition ratios of FMR molecules between H_2_O and CH_2_Cl_2_. (c) Normalized PL spectra of FMR molecules in water. (d) PL spectra of **C**_**10**_ **‐FMR** in water before and after the addition of DOPC. (e) Fluorescence enhancement ratios of FMR molecules in DOPC solution to those in H_2_O. (f) Dynamic tracking of the plasma membrane during PEG‐induced fusion of CRBC cells using **C**_**10**_ **‐FMR**. Reproduced from Ref.^[^ ^37^ ^]^ with permission. Copyright (2024), Elsevier. CRBC, chicken red blood cell; FMR, fluorescent molecular rotors; PEG, polyethylene glyco; PL, photoluminescence.

Cell membrane fusion plays a critical role in various biological processes, including cell growth, division, differentiation, and signaling.^[36]^ Monitoring this fusion can enhance our understanding of intracellular mechanisms and regulation. Currently, small molecule fluorescent probes enable long‐term, non‐invasive, and real‐time observation of cell membrane fusion in situ. By adjusting the lengths of side chains in fluorescent dyes, researchers can modulate cell permeability and dye anchoring to the plasma membrane. Yao et al. developed a series of fluorescent molecular rotors (FMR) dyes based on a classical twisted intramolecular charge transfer backbone (Figure 12a), that is, amino‐styryl‐pyridinium with two positive charges and varying side chain lengths.^[37]^ These amphiphilic FMR dyes readily integrate into the plasma membrane bilayer. Dyes with longer side chains have stronger anchoring abilities to the low‐polarity plasma membrane (Figure 12b), with fluorescence emission peaks primarily around 650 nm, suitable for long‐term imaging of living cells (Figure 12c). The **C**_**10**_ **‐FMR** dye shows weak fluorescence in water, but upon adding 1,2‐Dioleoyl‐sn‐glycero‐3‐phosphocholine (DOPC), its fluorescence intensity increases by 85.5‐fold. This indicates that the fluorescence of FMR dyes can be activated by the plasma membrane due to changes in polarity and movement restrictions in their environment (Figure 12d). Notably, the fluorescence enhancement ratio increased with side chain length, ranging from 4.1 for **C**_**1**_ **‐FMR** to 114.7 for **C**_**12**_ **‐FMR** (Figure 12e). This trend can be attributed to the increased lipophilicity of the FMR dyes, allowing those with longer chains to penetrate deeper into DOPC and exhibit higher fluorescence enhancement. Among the series, **C**_**10**_ **‐FMR** outperformed the others in imaging specificity and duration, making it particularly suitable for real‐time monitoring of plasma membrane‐related physiological processes. The successful tracking of polyethylene glyco‐induced fusion of chicken red blood cells (CRBCs) using **C**_**10**_ **‐FMR** illustrated its efficacy (Figure 12f); initially, cells approach and adhere, sharing portions of their plasma membranes, ultimately merging into a single cell. This research confirms that tuning side chain lengths can enhance the membrane dye permeability, thus providing a strategic approach for designing fluorescent dyes for three‐dimensional and long‐term dynamic tracking of plasma membranes across various animal cells.

**FIGURE 13:**
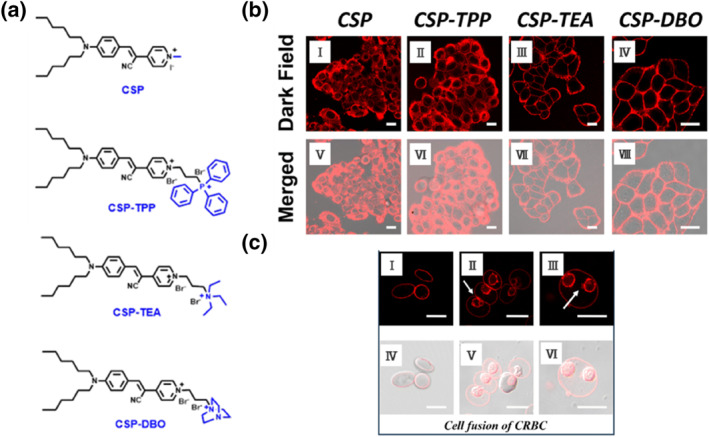
(a) Chemical structures of **CSP**, **CSP‐TPP**, **CSP‐TEA**, and **CSP‐DBO**. (b) CLSM images of HeLa cells stained with (I and V) **CSP** dyes, (II and VI) **CSP‐TPP**, (III and VII) **CSP‐TEA**, (IV and VIII) **CSP‐DBO**. (c) Membrane dynamics during cell fusion of CRBCs treated with PEG. Reproduced from Ref.^[^ ^38^ ^]^ with permission. Copyright (2024), Royal Society of Chemistry. CLSM, confocal laser scanning microscopy; CRBCs, chicken red blood cells; PEG, polyethylene glyco.

Currently, many highly specific fluorescent dyes for plasma membranes utilize lipophilic long alkyl chains as targeting units. However, the binding force through this mode is relatively weak. To address this, Zuo et al. proposed a module‐based universal design strategy for plasma membrane fluorescent dyes, comprising a central dye backbone module, a targeting module, an impermeable module, and a soluble module.^[38]^ Four fluorescent dye molecules with varying charge numbers and types were designed and synthesized, as illustrated in Figure 13a. Using cyano‐substituted styrylpyridinium as the backbone, two six‐carbon alkyl chains were attached at one end as targeting groups, while different anchoring groups−neutral methyl and alkyl triphenylphosphines (TPP), alkyl triethylammoniums (TEA), and alkyl 1,4‐diazabicycloalkanes (DBO)−were incorporated. These cyano‐styrylpyridine (**CSP**) dye molecules exhibit similar emission wavelengths and target‐activated fluorescence properties, yet they demonstrate distinct plasma membrane anchoring capabilities during plasma membrane imaging. HeLa cells were used to test whether the charge number and type of dye molecules influence their ability to anchor to the plasma membrane (Figure 13b). **CSP** and **CSP‐TPP** exhibited limited internalization, whereas **CSP‐TEA** and **CSP‐DBO** labeled the plasma membrane specifically and clearly, with **CSP‐DBO** providing more uniform staining than **CSP‐TEA**. Following incubation with **CSP‐DBO**, the plasma membranes of CRBCs were distinctly labeled (Figure 13c (I and IV)), allowing observation of the cell fusion process. Initially, multiple cells approached and adhered to each other. As fusion progressed, their plasma membranes merged in specific regions while keeping the cytoplasm separate (Figure 13c (II and V)). Eventually, the gap between the cells disappeared, leading to cytoplasmic fusion and encapsulation of multiple nuclei within a single cell (Figure 13c (III and VI)). This proposed design framework is expected to enhance the development of fluorescent dyes for long‐term imaging of cytoplasmic membranes, significantly expanding applications in important physiological processes at both cellular and in vivo levels, and paving the way for novel 4D imaging tools.

**FIGURE 14:**
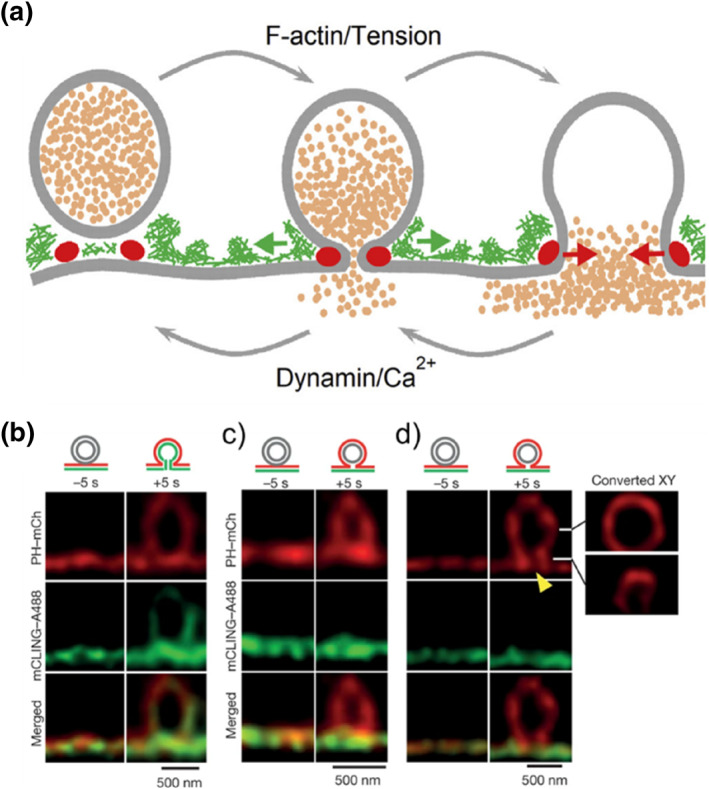
(a) Visualization of membrane pores in live cells reveals the dynamic pore processes that control fusion and endocytosis. Reproduced from Ref.^[^ ^41^ ^]^ with permission. Copyright (2018), Cell Press. (b)–(d) Super‐resolution stimulated emission depletion *XZ* images showing tagged phospholipase Cδ1 PH domain (PH)‐mCherry and **mCLING‐A488**. Panel (b) represents a typical  Ω‐profile from 53 profiles, while panels (c) and (d) show PH only Ω‐profiles with undetectable gaps and a detectable gap at the neck region (d). Reproduced from Ref.^[^ ^42^ ^]^ with permission. Copyright (2016), Springer Nature. STED, stimulated emission depletion.

Short peptides possess excellent properties such as biocompatibility, ease of synthesis, amphiphilicity, and ease of modification, making them increasingly attractive as recognition elements for anchoring plasma membranes.^[39]^ The membrane‐binding fluorophore‐cysteine‐lysine‐palmitoyl group (mCLING) probe^[40]^ is a novel commercially available dye consisting of an octapeptide with one cysteine and seven lysines, one of which is attached to a palmitoyl tail. This structure allows the cysteine to bridge to the fluorophore via a maleimide moiety. For example, Shin et al. utilized the mCLING probe to observe dynamic behaviors of fusion pores—such as opening, expansion, contraction, and closure—in live neuroendocrine cells (Figure 14a).^[41]^ This observation supports the fusion pore hypothesis and establishes a dynamic pore theory for living cells, explaining the processes of fusion, fission, and their regulation. Additionally, the study employed the mCLING probe to monitor how secretory vesicles fuse and divide with the cell membrane in real time, illustrating its crucial role in membrane fusion and division (Figure 14b–d).^[42]^

**FIGURE 15:**
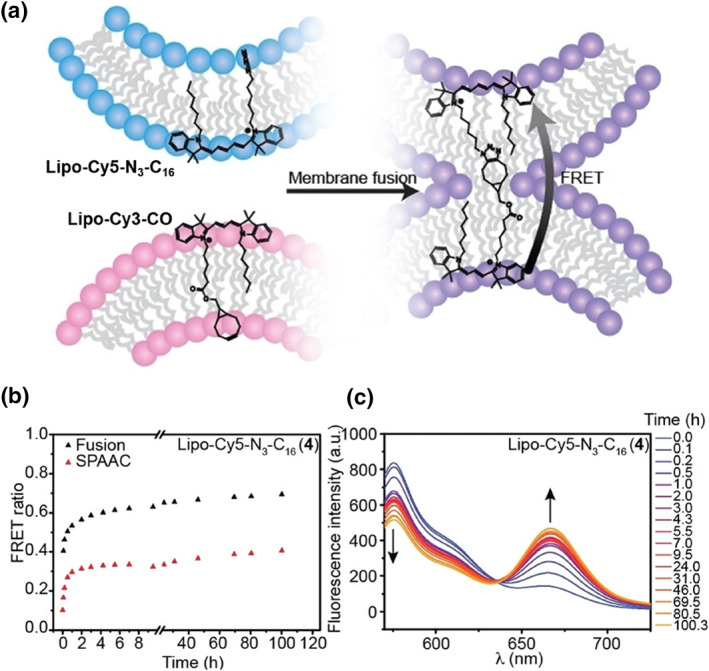
(a) Schematic diagram of the liposome fusion assay. Fusion of liposomes containing FRET donor or acceptor dyes results in an increase in FRET following SPAAC. (b) Kinetic analysis of FRET ratios, comparing liposome fusion (black symbols) to chemical conjugation post‐cleavage (red symbols). (c) FRET profiles over time following liposome disruption with ethanol. Reproduced from Ref.^[^ ^43^ ^]^ with permission. Copyright (2024), John Wiley and Sons. FRET, Förster resonance energy transfer; SPAAC, strain‐promoted azide‐alkyne cycloaddition.

In addition to conventional single‐molecule fluorescent probes, accurate monitoring of cell membrane fusion can be achieved using Förster resonance energy transfer (FRET) dyes functionalized with azide and strained alkynes, thus minimizing false signals. Jumeaux et al. synthesized novel lipophilic dyes featuring strain‐promoted azide‐alkyne cycloaddition (SPAAC) reactive handles, centered on Cy3 and Cy5, which exhibit FRET when conjugated within lipid bilayers (Figure 15a).^[43]^ The importance of hydrophobicity for the retention of **Lipo‐Cy5‐N**_**3**_ **‐C**_**1**6_ in the membrane and the fusion dependence of SPAAC product formation was further demonstrated through synthetic design and atomistic simulations. The kinetics of the SPAAC reaction within the lipid bilayer were investigated by mixing dsDNA‐modified liposomes, **Lipo‐Cy3‐CO** liposomes, and **Lipo‐Cy5‐N**_**3**_ **‐C**_**16**_ liposomes with aliquots taken at various time intervals (Figure 15b,c). FRET spectra were measured before and after liposome disruption to track the kinetics of liposome fusion and chemical conjugation. The kinetics of SPAAC is closely correlated with liposome fusion kinetics, though a decrease in the FRET ratio was observed. This decrease was attributed to interactions between multiple donor and acceptor dyes within the confined space of the lipid membrane, enhancing FRET efficiency. Upon disruption, FRET signaling was limited to interactions between single chemically conjugated donor‐acceptor pairs. This study characterizes, for the first time, the SPAAC response between diffusing molecules within liposome membranes, illustrating liposome fusion as a trigger for bilayer confinement chemical reaction and highlighting potential applications for novel biomarkers and biosensing strategies using liposome fusion events.

## 5 CONCLUSION

To date, fluorescent probe technology has become an indispensable tool in biological research, particularly for in situ and real‐time imaging of physiological and pathological processes.^[44]^ The dynamic and heterogeneous nature of cell membranes poses significant challenges for studying these processes.^[45]^ Small‐molecule fluorescent probes have emerged as vital tools in cell membrane research due to their ease of synthesis and modifiability. They have been crucial in examining the dynamic dissociation and reorganization of membrane microdomains,^[46]^ imaging cell division and fusion,^[47]^ providing high‐fidelity imaging of organelle membranes, and developing super‐resolution imaging techniques^[48]^ to capture the fine structure of cell membranes.

We anticipate that the development of small‐molecule fluorescent probes with higher brightness and longer wavelengths will enhance dynamic imaging of cell membranes. However, several challenges remain:

1) **Solubility of Fluorescent Probes**: Effective membrane‐targeted probes must possess both lipophilic and hydrophilic properties due to the lipid‐based composition of cell membranes and the aqueous external environment. This dual solubility requirement necessitates a more sophisticated probe design.
2) **Selectivity for Organelle Membranes**: Probes must penetrate the outer plasma membrane to target specific internal organelle membranes, which vary significantly in composition and structure. For example, mitochondrial membranes differ significantly between the outer and inner layers.^[49]^ Achieving precise labeling of specific organelles within the complex cellular environment remains a significant challenge.
3) **Dynamic Imaging of Membrane Processes**: Real‐time imaging of dynamic cellular membrane processes requires probes to maintain their association with the membrane during continuous microdomain dissociation and reorganization. This dynamic nature often leads to the loss of fluorescent signals as probes detach from the membrane, underscoring the need for enhanced membrane‐targeting capabilities for prolonged imaging.
4) **Activatable fluorescent probes**: The cell membrane serves as a protective barrier, shielding cells from external invaders while facilitating signal recognition and transduction. It is thus closely involved in numerous critical physiological processes, including phagocytosis, adhesion, and migration. Furthermore, dynamic processes such as protein rearrangement and post‐translational modifications,^[50]^ including glycosylation and phosphorylation, significantly impact the biological functions of the plasma membrane. Real‐time, in situ tracking of these dynamic events is essential for understanding and regulating key physiological functions at the cell membrane. However, there are few reports on activatable fluorescent probes^[51]^ capable of detecting dynamic processes on the cell membrane due to its inherent dynamics and heterogeneity. With the advancement of super‐resolution imaging techniques and the incorporation of optical,^[52]^ electrical, thermal, magnetic, and chemical stimuli into membrane probe design, future developments will undoubtedly accelerate progress in cell membrane research.
5) **Resolution**: Distinguishing between membrane microdomains, typically ranging from 20 to 100 nm,^[53]^ remains challenging even with current super‐resolution imaging techniques, making clear differentiation difficult.

Looking ahead, advancements in organic synthesis and high‐performance imaging instruments will undoubtedly lead to the development of more sophisticated fluorescent probes, enabling deeper and more comprehensive studies of cell membrane‐related processes. This progress will ultimately drive the detection and treatment of membrane‐related diseases.

## CONFLICT OF INTEREST STATEMENT

The authors declare no conflicts of interest.
